# The effect of anxiety and depression on progression of glaucoma

**DOI:** 10.1038/s41598-021-81512-0

**Published:** 2021-01-19

**Authors:** Da Young Shin, Kyoung In Jung, Hae Young Lopilly Park, Chan Kee Park

**Affiliations:** Department of Ophthalmology, Seoul St. Mary’s Hospital, College of Medicine, The Catholic University of Korea, 222 Banpo-daero, Seocho-gu, Seoul, 06591 Republic of Korea

**Keywords:** Diseases, Risk factors

## Abstract

Glaucoma is considered a chronic disease that requires lifelong management. Chronic diseases are known to be highly associated with psychological disturbances such as depression and anxiety. There have also been many studies on association between anxiety or depression and glaucoma. The majority of these studies explained that the glaucoma diagnosis causes anxiety or depression. However, It is also necessary to evaluate whether the psychological disturbance itself affect glaucoma. Therefore, we investigated the association of anxiety and depression with glaucoma progression, and elucidate mechanisms underlying that. We included 251 eyes with open angle glaucoma who were followed up for at least 2 years in this retrospective case–control study. The Beck Anxiety Inventory (BAI) and Beck Depressive Inventory-II (BDI-II) were used to assess anxiety and depression in glaucoma patients. Patients were classified into groups (high-anxiety group; HA-G, low-anxiety group; LA-G, high-depression group; HD-G, low-depression group; LD-G) according to their score on the BAI or BDI-II (separately). In logistic regression analysis, disc hemorrhage, peak intraocular pressure (IOP) and RNFL thickness loss rate were significantly associated with high anxiety (*p* = 0.017, *p* = 0.046, *p* = 0.026). RNFL thinning rate and disc hemorrhage were significant factors associated with anxiety in multivariate models (*p* = 0.015, *p* = 0.019). Multivariate linear regression analysis showed a significant positive correlation between the rate of RNFL thickness loss and BAI score (B = 0.058; 95% confidential interval = 0.020–0.097; *p* = 0.003), and RNFL loss and IOP fluctuation (B = 0.092; 95% confidential interval = 0.030–0.154; *p* = 0.004). For the depression scale, visual field mean deviation and heart rate variability were significantly associated with high depression in multivariate logistic regression analysis (*p* = 0.003, *p* = 0.006). We suggest that anxiety increase the risk of glaucoma progression and they are also associated with IOP profile and disc hemorrhage.

Glaucoma is optic neuropathy characterized by progressive loss of retinal ganglion cells^1^. There is currently no effective treatment for ganglion cell degeneration, and the treatment of glaucoma is focused on preventing progression^2^. Therefore, glaucoma is considered a chronic disease that requires lifelong management^3^.

It is known that chronic diseases are associated with psychological disturbances such as depression and anxiety^4,5^. In glaucoma, there have been many studies about anxiety or depression, which have reported that the prevalence of anxiety or depression is high in patients with glaucoma^6–8^. The majority of these studies explained that the high prevalence of anxiety and/or depression is the consequence of being diagnosed with glaucoma, and results from the fear of potential blindness, heavy economic burden and impaired daily activity^9,10^. Whereas most studies have documented the disease as contributing to anxiety/depression, several lines of evidence now show that negative emotions such as anxiety/depression, are also a risk factor for physical illness^11–14^. They reported that anxiety or depression may speed the development of disease such as cardiovascular disorders, and worsen diseases such as gastrointestinal or respiratory disorders^13–16^. Recently, there was a study showing a patient with glaucoma suspect who have history of anxiety or depression developed more glaucoma, suggesting that emotional stress itself may have effect on glaucoma^17^.

Anxiety and depression are reactions to stress and are thought to arise in the amygdala^18^. These emotional responses evoke secretion of neurotransmitters and stimulate the autonomic nervous system (ANS), which affects multiple organs^19^. The ANS, which can be affected by emotions, is also important in the development or progression of glaucoma^20–22^.

Many studies, including randomized control trials, have investigated risk factors for glaucoma progression^23–25^. Based on their results, risk factors associated with progression include old age, increased mean or peak intraocular pressure (IOP), greater IOP fluctuation, the presence of disc hemorrhage (DH), myopia, and low or high blood pressure (BP)^23–27^. These factors largely can be classified into mechanical (IOP) and vascular (DH, BP) caegories^27^.

The purpose of the present investigation was to evaluate whether anxiety and depression affect glaucoma progression, and to elucidate mechanisms underlying that association through mechanical and vascular factors.

## Results

The patients’ baseline characteristics are listed in Table 1. The average follow-up period was 62.8 ± 32.1 (mean ± standard deviation) months. All patients were taking some form of glaucoma medication; 183 (72.9%) had been prescribed prostaglandins. The mean BAI score was 6.0 ± 4.4 (range, 0–22). Among 251 glaucoma patients, 209 (83.3%) were in the LA-G (range, 0–10) and 44 (16.7%) were in the HA-G (range, 11–22). The results of comparisons between the two groups divided according to BAI scale are summarized in Tables 2 and 3. The incidence of DH was higher in the HA-G than LA-G (3.3% vs 11.9%, *p* = 0.018). Mean IOP (13.76 ± 3.00 vs 14.76 ± 3.02, *p* = 0.049) and peak IOP (17.43 ± 4.12 vs 18.86 ± 4.32, *p* = 0.043) were higher in the HA-G than the LA-G (Table 2). The rate of RNFL thinning during follow-up in the HA-G (− 1.96 ± 2.23 µm/year) was faster than in the LA-G (− 0.68 ± 1.39 µm/year, *p* = 0.021, Table 2). The results of logistic regression are listed in Table 3. RNFL thickness loss rate (OR = 1.69, 95% CI = 1.20–2.38, *p* = 0.003) and DH (OR = 6.79, 95% CI = 1.48–31.08, *p* = 0.014) were significant factors associated with anxiety in multivariate model. We investigated which questions were associated with the rate of RNFL loss. Multiple questions (numbness or tingling, feeling hot, wobbliness in legs, dizzy or lightheaded, heart pounding/racing, unsteady, terrified or afraid, face flushed) were shown to be associated with RNFL loss rate (Table 4).

**Table 1:** Baseline demographics of patients with glaucoma.

Variables	Open angle glaucoma, 251 eyes
Age, y	53.23 ± 13.03
Sex, male/female	107/144
Hypertension, n (%)	47(18.7%)
Diabetics, n (%)	10(4.0%)
CCT, μm	537.23 ± 46.37
Axial length, mm	25.31 ± 1.87
PPA/Disc area, per 1 µm larger (%)	44.18 ± 51.55
Tilt ratio	1.20 ± 0.21
Torsion ratio	88.50 ± 9.73
VF MD, dB	− 4.38 ± 5.30
VF PSD, dB	4.51 ± 3.61
Follow-up duration (month)	62.78 ± 32.09
Mean IOP	13.93 ± 3.00
Peak IOP	17.67 ± 4.18
IOP fluctuation	5.67 ± 3.37
Mean blood pressure	94.13 ± 11.68
Disc hemorrhage	12(4.8%)
Heart rate variability	35.26 ± 21.28
Beck anxiety inventory score	6.00 ± 5.45
Beck depression inventory-II score	8.81 ± 6.48

CCT, central corneal thickness; PPA, peripapillary atrophy; VF, visual field; MD, mean deviation; PSD, pattern standard deviation; IOP, intraocular pressure.

Continuous data are mean ± mean standard deviation unless otherwise indicated.

**Table 2:** Comparison of the demographics and test results between two groups divided according to the Beck Anxiety Inventory (BAI) scale.

Variables	Low anxiety group (LA − G), 209 eyes	High anxiety group (HA − G), 42 eyes	*P* value
Age, y	53.05 ± 12.16	54.14 ± 16.87	0.691*
Sex, male/female	89/120	18/24	0.974^†^
Hypertension, n (%)	37(17.7%)	10(23.8%)	0.355^†^
Diabetics, n (%)	8(3.8%)	2(4.8%)	0.778^†^
CCT, µm	539.12 ± 48.06	527.22 ± 34.98	0.184*
Axial length, mm	25.29 ± 1.94	25.38 ± 1.47	0.764*
PPA/Disc area, per 1 µm larger (%)	46.01 ± 54.13	35.27 ± 35.66	0.219^†^
Tilt ratio	1.20 ± 0.22	1.20 ± 0.16	0.978^†^
Torsion ratio	88.97 ± 9.69	87.33 ± 7.59	0.304^†^
VF MD, dB	− 4.55 ± 5.49	− 3.54 ± 4.18	0.206*
VF PSD, dB	4.66 ± 3.70	3.78 ± 3.12	0.148*
VF MD progression rate, dB/year	− 0.55 ± 0.58	− 0.76 ± 0.73	0.310*
RNFL thickness loss rate, µm/year	− 0.68 ± 1.39	− 1.96 ± 2.23	0.021*
Follow-up duration (month)	63.85 ± 30.92	56.95 ± 38.26	0.379*
Mean IOP	13.76 ± 3.00	14.76 ± 3.02	0.049*
Peak IOP	17.43 ± 4.12	18.86 ± 4.32	0.043*
IOP fluctuation	5.50 ± 3.41	6.50 ± 3.08	0.080*
Mean blood pressure	94.48 ± 12.503	92.45 ± 6.21	0.264*
Disc hemorrhage	7(3.3%)	5(11.9%)	**0.018**^†^
Heart rate variability	35.09 ± 18.99	42.10 ± 29.86	0.150*

CCT, central corneal thickness; PPA, peripapillary atrophy; VF, visual field; MD, mean deviation; PSD, pattern standard deviation; RNFL, retinal nerve fiber layer; IOP, intraocular pressure.

*The comparison was performed using independent samples t-test.

^†^The comparison was performed using a chi-squared test.

Continuous data are mean ± mean standard deviation unless otherwise indicated.

Statistically significant values appear in boldface.

**Table 3:** Factors associated with the Beck Anxiety Inventory (BAI) scores in patients with glaucoma. Logistic Regression Analysis of BAI score.

Variables	Univariate model		Multivariate model	
	Odds ratio, 95% CI	*P* value	Odds ratio, 95% CI	*P* value
Age, y	1.01, 0.98–1.03	0.619		
Sex, male/female	0.99, 0.51–1.93	0.989		
Hypertension, n (%)	1.45, 0.66–3.21	0.357		
Diabetics, n (%)	1.26, 0.26–6.14	0.778		
CCT, µm	0.99, 0.99–1.00	0.185		
Axial length, mm	1.03, 0.85–1.24	0.802		
PPA/Disc area, per 1 µm larger (%)	0.59, 0.25–1.37	0.220		
Tilt ratio	0.87, 0.17–4.42	0.867		
Torsion ratio	0.98, 0.95–1.02	0.303		
VF MD, dB	1.04, 0.97–1.12	0.262		
VF PSD, dB	0.93, 0.84–1.03	0.151		
VF MD progression rate, dB/year	1.63, 0.63–4.18	0.314		
RNFL thickness loss rate, µm/year	1.66, 1.19–2.33	**0.003**	1.69, 1.20–2.38	**0.003**
Follow-up duration (month)	0.99, 0.98–1.01	0.377		
Mean IOP	1.12, 1.00–1.25	0.051		
Peak IOP	1.08, 1.00–1.17	**0.046**	1.00, 0.87–1.15	0.991
IOP fluctuation	1.08, 0.99–1.19	0.084		
Mean blood pressure	0.98, 0.94–1.03	0.489		
Disc hemorrhage	3.90, 1.18–12.95	**0.026**	6.79, 1.48–31.08	**0.014**
Heart rate variability	1.01, 1.00–1.03	0.059		

CI, confidence interval; CCT, central corneal thickness; PPA, peripapillary atrophy; VF, visual field; MD, mean deviation; PSD, pattern standard deviation; RNFL, retinal nerve fiber layer; IOP, intraocular pressure.

Variables with *p* < 0.05 were included in the multivariate analysis.

Statistically significant values are shown in bold.

**Table 4:** The questions on the BECK anxiety inventory (BAI) associated with RNFL thickness loss.

Variables	RNFL thickness progression rate, um/year		*P* value
	Score 0	Score 1–3	
**Q1. Numbness or tingling**	0.63 ± 1.47	1.49 ± 1.80	**0.006**
**Q2. Feeling hot**	0.70 ± 1.56	1.34 ± 1.67	**0.040**
**Q3. Wobbliness in legs**	0.70 $\pm 1.37$	2.56 $\pm 2.62$	**0.033**
Q4. Unable to relax	0.75 $\pm 1.45$	1.20 $\pm 1.95$	0.158
Q5. Fear of worst happening	0.89 $\pm 1.38$	0.83 $\pm 2.10$	0.851
**Q6. Dizzy or lightheaded**	0.62 $\pm 1.45$	1.19 $\pm 1.75$	**0.042**
**Q7. Heart pounding/racing**	0.47 $\pm 1.46$	1.34 $\pm 1.65$	**0.002**
**Q8. Unsteady**	0.66 $\pm 1.42$	1.24 $\pm 1.85$	**0.047**
**Q9. Terrified or afraid**	0.62 $\pm 1.41$	1.54 $\pm 1.90$	**0.011**
Q10. Nervous	0.82 $\pm 1.76$	0.94 $\pm 1.41$	0.661
Q11. Feeling of choking	0.81 $\pm 1.60$	1.29 $\pm 1.64$	0.250
Q12. Hands trembling	0.75 $\pm 1.39$	2.25 $\pm 2.91$	0.121
Q13. Shaky/unsteady	0.72 $\pm 1.38$	2.42 $\pm 2.71$	0.053
Q14. Fear of losing control	0.75 $\pm 1.36$	3.51 $\pm 3.54$	0.114
Q15. Difficulty in breathing	0.72 $\pm 1.35$	0.09 $\pm 2.69$	0.070
Q16. Fear of dying	0.84 $\pm 1.61$	1.96 $\pm 1.38$	0.171
Q17. Scared	0.78 $\pm 1.49$	1.04 $\pm 1.80$	0.400
Q18. Indigestion	0.73 $\pm 1.42$	1.08 $\pm 1.85$	0.234
Q19. Faint/lightheaded	0.86 $\pm 1.62$	0.27 $\pm 0.25$	0.530
**Q20. Face flushed**	0.69 $\pm 1.41$	1.66 $\pm 2.12$	**0.038**
Q21. Hot/cold sweats	0.82 $\pm 1.66$	1.08 $\pm 1.39$	0.423

The comparison was performed using independent samples t-test.

Statistically significant values are shown in bold.

Continuous data are mean ± mean standard deviation unless otherwise indicated.

The mean BDI-II score was 8.5 ± 6.4 (range, 0–32). Among 251 patients with open angle glaucoma, 211 (84.1%) were in the LD-G (range, 0–14) and 40 (15.9%) in the HD-G (range, 15–32). The results of comparisons between the two groups divided according to BDI-II scores are shown in Table 5. In BDI-II analysis, the HD-G showed worse VF mean deviation (MD) (− 7.30 ± 7.68 dB) than did the LD-G (− 3.81 ± 4.50 dB, *p* =  < 0.001, Table 5). Heart rate variability was significantly higher in the HD-G (44.77 ± 36.27) than the LD-G (34.08 ± 16.28, *p* = 0.001, Table 5). MD of VF (OR = 0.91, 95% CI = 0.86–0.97, *p* = 0.003) and Heart rate variability (OR = 1.02, 95% CI = 1.01–1.04, *p* = 0.006) were significant factors associated with BDI-II in both the univariate and multivariate models (Table 6). We investigated which questions are associated with the worse MD in VF. Patients with punishment feelings (− 4.02 ± 4.94 vs − 5.78 ± 6.35, *p* = 0.036) or self-dislike (− 3.83 ± 4.19 vs − 5.59 ± 7.06, *p* = 0.047) showed worse MD than did patients without such feelings (Table 7).

**Table 5:** Comparison of the demographics and test results between two groups divided according to the Beck depression inventory-II (BDI-II) scale.

Variables	Low depression group, (LD-G), 211 eyes	High depression group, (HD-G), 40 eyes	*P* value
Age, y	52.67 ± 12.02	56.20 ± 17.34	0.224*
Sex, male/female	93/118	14/26	0.287^†^
Hypertension, n (%)	39(18.5%)	8(20.0%)	0.822^†^
Diabetics, n (%)	8(3.8%)	2(5.0%)	0.720^†^
CCT, µm	539.66 $\pm 48.31$	523.33 $\pm 30.10$	0.109*
Axial length, mm	25.30 $\pm 1.86$	25.36 $\pm 1.96$	0.847*
PPA/Disc area, per 1 µm larger (%)	45.41 $\pm 53.98$	37.86 $\pm 36.40$	0.398*
Tilt ratio	1.21 $\pm 0.22$	1.18 $\pm 0.16$	0.242*
Torsion ratio	88.93 $\pm 14.51$	91.04 $\pm 10.35$	0.699*
VF MD, dB	− 3.81 $\pm 4.50$	− 7.30 $\pm 7.68$	< 0.001*
VF PSD, dB	4.31 $\pm 3.52$	5.54 $\pm 3.94$	0.053*
VF MD progression rate, dB/year	− 0.56 $\pm 0.59$	− 0.67 $\pm 0.62$	0.539*
RNFL thickness loss rate, µm/year	− 0.76 $\pm 1.42$	− 1.45 $\pm 2.33$	0.074*
Follow-up duration (month)	63.02 $\pm$ 31.16	61.57 $\pm$ 37.36	0.851*
Mean IOP	13.93 $\pm$ 2.96	13.93 $\pm$ 3.25	0.993*
Peak IOP	17.66 $\pm 4.27$	17.70 $\pm 3.76$	0.960*
IOP fluctuation	5.63 $\pm 3.51$	5.88 $\pm 2.54$	0.675*
Mean blood pressure	94.89 $\pm 12.26$	90.42 $\pm 7.45$	0.102*
Disc hemorrhage	11(5.2%)	2(2.5%)	0.461^†^
Heart rate variability	34.08 ± 16.28	47.77 ± 36.27	0.001*

CCT, central corneal thickness; PPA, peripapillary atrophy; VF, visual field; MD, mean deviation; PSD, pattern standard deviation; RNFL, retinal nerve fiber layer; IOP, intraocular pressure.

Statistically significant values appear in boldface.

*The comparison was performed using independent samples t-test.

^†^The comparison was performed using a chi-squared test.

Continuous data are mean ± mean standard deviation unless otherwise indicated.

**Table 6:** Factors associated with the Beck Depression Inventory (BDI-II) scale in patients with glaucoma.

Variables	Univariate model		multivariate model	
	Odds ratio, 95% CI	*P* value	Odds ratio, 95% CI	*P* value
Age, y	1.02, 0.99–1.05	0.118		
Sex, male/female	1.46, 0.72–2.96	0.289		
Hypertension, n (%)	1.10, 0.47–2.58	0.822		
Diabetics, n (%)	1.34, 0.27–6.53	0.721		
CCT, µm	0.99, 0.98–1.00	0.110		
Axial length, mm	1.02, 0.84–1.23	0.847		
PPA/Disc area, per 1 µm larger (%)	0.71, 0.32–1.57	0.379		
Tilt ratio	0.42, 0.07–2.47	0.337		
Torsion ratio	1.01, 0.98–1.05	0.502		
VF MD, dB	0.91, 0.86–0.96	** < 0.001**	0.91, 0.86–0.97	**0.003**
VF PSD, dB	1.09, 1.00–1.19	0.051		
VF MD progression rate, dB/year	1.32, 0.54–3.22	0.536		
RNFL thickness loss rate, µm/year	1.30, 0.97–1.73	0.079		
Follow-up duration (month)	0.99, 0.98–1.01	0.849		
Mean IOP	1.00, 0.89–1.12	0.993		
Peak IOP	1.00, 0.92–1.09	0.960		
IOP fluctuation	1.02, 0.93–1.13	0.674		
Mean blood pressure	0.94, 0.92–1.01	0.103		
Disc hemorrhage	0.47, 0.06–3.71	0.471		
Heart rate variability	1.02, 1.01–1.04	**0.001**	1.02, 1.01–1.04	**0.006**

CI, confidence interval; CCT, central corneal thickness; PPA, peripapillary atrophy; VF, visual field; MD, mean deviation; PSD, pattern standard deviation; RNFL, retinal nerve fiber layer; IOP, intraocular pressure.

Variables with *p* < 0.05 were included in the multivariate analysis.

Statistically significant values appear in boldface.

Continuous data are mean ± mean standard deviation unless otherwise indicated.

**Table 7:** The questions on the BECK Depression Inventory-II (BDI-II) associated with visual field mean deviation.

Variables	Mean deviation of visual field, dB		*P* value
	Score 0	Score 1–3	
Q1. Sadness	− 4.14 ± 5.02	− 4.65 ± 5.61	0.454
Q2. Pessimism	− 3.93 $\pm 4.22$	− 4.56 $\pm 5.67$	0.405
Q3. Past Failure	− 4.08 $\pm 4.64$	− 5.30 $\pm 6.90$	0.206
Q4. Loss of Pleasure	− 4.53 $\pm 5.39$	− 4.14 $\pm 5.16$	0.568
Q5. Guilty Feelings	− 4.47 $\pm 4.95$	− 4.22 $\pm 5.85$	0.851
**Q6. Punishment Feelings**	** − 4.02 ± 4.94**	** − 5.78 ± 6.35**	**0.036**
**Q7. Self-Dislike**	** − 3.83 ± 4.19**	** − 5.59 ± 7.06**	**0.047**
Q8. Self-Criticalness	− 3.98 $\pm 4.54$	− 4.74 $\pm 5.89$	0.263
Q9. Suicidal Thoughts or Wishes	− 4.33 $\pm 5.38$	− 4.67 $\pm 4.82$	0.726
Q10. Crying	− 4.43 $\pm 5.29$	− 3.91 $\pm 5.48$	0.663
Q11. Agitation	− 4.27 $\pm 5.09$	− 4.60 $\pm 5.71$	0.642
Q12. Loss of Interest	− 4.22 $\pm 4.89$	− 4.87 $\pm 6.38$	0.409
Q13. Indecisiveness	− 3.99 $\pm 5.03$	− 5.09 $\pm 5.72$	0.137
Q14. Worthlessness	− 4.49 $\pm 5.24$	− 4.13 $\pm 5.44$	0.625
Q15. Loss of Energy	− 4.21 $\pm 4.96$	− 4.52 $\pm 5.57$	0.647
Q16. Changes in Sleeping Pattern	− 4.43 $\pm 4.89$	− 4.34 $\pm 5.62$	0.171
Q17. Irritability	− 4.24 $\pm 4.97$	− 4.47 $\pm 5.51$	0.743
Q18. Changes in Appetite	− 3.96 $\pm 4.59$	− 6.27 $\pm 7.52$	0.056
Q19. Concentration Difficulty	− 4.35 $\pm 5.25$	− 4.61 $\pm 5.81$	0.823
Q20. Tiredness or Fatigue	− 4.88 $\pm 5.29$	− 4.01 $\pm 5.28$	0.201
Q21. Loss of Interest in Sex	− 3.76 $\pm 4.74$	− 4.81 $\pm 5.63$	0.113

The comparison was performed using independent samples t-test.

Statistically significant values are shown in bold.

Continuous data are mean ± mean standard deviation unless otherwise indicated.

**Figure 1:**
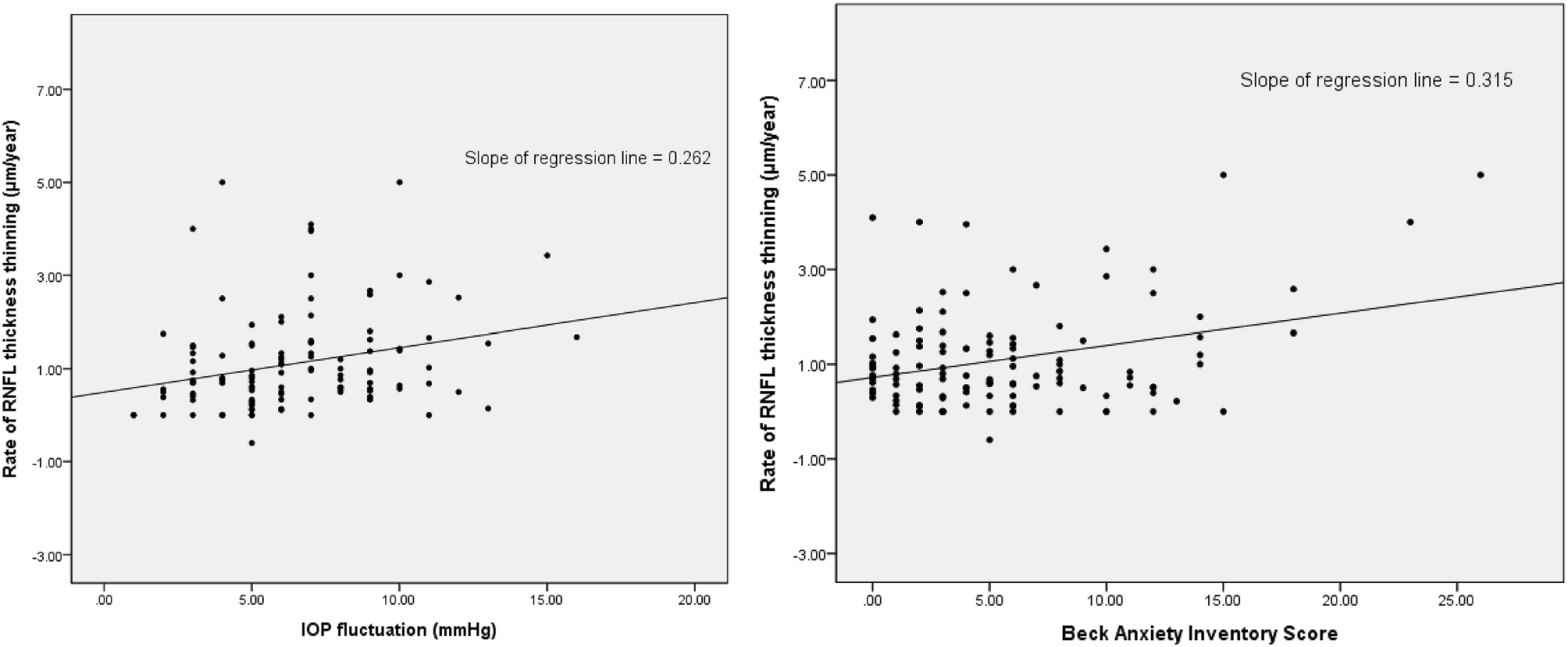
Scatter plot showing the relationships between the rate of RNFL thinning and IOP fluctuations (left) and Beck Anxiety Inventory scores (right).

Parameters related to the rate of RNFL thinning were evaluated by linear regression analyses. BAI score (B = 0.058, 95% confidential interval = 0.020–0.097, *p* = 0.003) and IOP fluctuation (β = 0.092, 95% confidential interval = 0.030–0.154, *p* = 0.004) were significantly related to the rate of RNFL thinning, based on multivariate analyses (Table 8). The relationships between RNFL thinning rates, and BAI scores and IOP fluctuations are shown in Fig. 1. The slope of the linear fit was positive for the rate of RNFL loss against both BAI score and IOP fluctuation.

**Table 8:** Regression analysis of factors associated with the RNFL thickness loss rate.

RNFL thickness loss rate	Univariate model				Multivariate model			
	B	β	95% CI	*P* value	B	β	95% CI	*P* value
Age, y	− 0.003	− 0.035	− 0.022 to 0.015	0.710				
Sex, male/female	− 0.029	− 0.013	− 0.426 to 0.369	0.546				
Hypertension, n (%)	− 0.288	− 0.089	− 0.878 to 0.302	0.336				
Diabetics, n (%)	− 0.018	− 0.003	− 1.109 to 1.073	0.998				
CCT, µm	0.001	0.057	− 0.146 to 0.105	0.747				
Axial length, mm	− 0.020	− 0.033	− 0.164 to 0.136	0.855				
PPA/Disc area (%)	0.102	0.034	− 0.454 to 0.658	0.717				
Tilt ratio	0.453	0.075	− 0.681 to 1.586	0.621				
Torsion ratio	− 0.015	− 0.137	− 0.035 to 0.005	0.150				
VF MD, dB	0.025	0.127	− 0.011 to 0.061	0.172				
VF PSD, dB	− 0.036	− 0.125	− 0.089 to 0.017	0.177				
VF MD rate, dB/year	− 0.008	− 0.027	− 0.090 to 0.074	0.847				
Mean IOP	0.003	0.011	− 0.050 to 0.056	0.907				
Peak IOP	0.047	0.116	− 0.004 to 0.098	0.072				
IOP fluctuation	0.096	0.262	0.031 to 0.161	**0.015**	0.092	0.251	0.030 to 0.154	**0.004**
Mean blood pressure	0.005	0.041	− 0.080 to 0.090	0.902				
Disc hemorrhage	0.075	0.015	− 0.859 to 1.008	0.874				
Heart rate variability	0.013	0.199	0.001 to 0.025	**0.031**	0.007	0.104	− 0.005 to 0.019	0.255
BAI score	0.068	0.315	0.030 to 0.105	**0.001**	0.058	0.272	0.020 to 0.097	**0.003**
BDI-II score	0.015	0.073	− 0.023 to 0.054	0.431				

B, non-standardized coefficient; β, standardized coefficient; CI, confidence interval; CCT, central corneal thickness; PPA, peripapillary atrophy; VF, visual field; MD, mean deviation; PSD, pattern standard deviation; RNFL, retinal nerve fiber layer; IOP, intraocular pressure; BAI, Beck Anxiety Inventory; BDI-II, Beck Depression Inventory-II.

Statistically significant values appear in boldface.

## Discussion

Depression and anxiety are highly prevalent in individuals with chronic disease^4,5^.The relationship between the chronic disease and depression/anxiety can be experienced as independent or inter-related (with either one causing the other)^12^.The majority of papers have reported that anxiety/depression is the consequence of being diagnosed with a chronic disease^12^. Diagnosis of chronic disease can cause anxiety/depression due to functional limitations, social isolation, loss of relationships, guilty feelings and anxiety about the future^9,10^.

Meanwhile, some other studies demonstrated that anxiety/depression led to or worsen chronic disease^11,12,28,29^. For example, high emotions cause high BP, depression causes heart disease, and persistent anxiety causes high blood sugar and diabetes^13,28^.

As glaucoma is a chronic disease, it has been the focus of many studies about anxiety and depression, and these studies have shown that the prevalence of anxiety and depression are high in glaucoma. The prevalence of anxiety in glaucoma patients has been reported to be in the range of 13.0–30%, and the prevalence of depression has been reported from 10.9 to 24.7%^5–9^. Most qualitative studies have reported that glaucoma patients interpret their disease as contributing to anxiety and/or depression^5–9^. However, as in other chronic disease studies, anxiety/depression could affect glaucoma^9,10^. Recently, Samuel et al.^17^ reported that a history of anxiety in glaucoma suspects was associated with developing glaucoma. This study did not investigate how anxiety might influence glaucoma progression. So we tried to clarify this mechanism by investigating the association between anxiety and well-known risk factors for glaucoma progression.

In our study, anxiety was significantly associated with the rate of RNFL thickness decline in patients with glaucoma (Tables 2 and 3). Although the statistical significance was borderline (*p* = 0.074, independent t-test), the rate of RNFL thinning was faster in the HD-G than the LD-G. These results suggest that not only is glaucoma a risk factor for anxiety/depression, but also that anxiety/depression could be a risk factor for glaucoma. The rate of VF progression was not significantly different between the low and high groups for either anxiety or depression. This is probably because the follow-up period was not long (5.23 ± 2.67 years), and the subjects had relatively early glaucoma (− 4.38 ± 5.30 dB). RNFL thinning or structural loss appears before functional VF defects, so OCT is more sensitive than VF testing for the detection of progression in early glaucoma^30^. Moreover, VF tests are difficult for some patients and are known to have increased variability^31^. For this reason, although the rate of VF progression did not show statistical significance, the rate of RNFL thickness loss is sufficient to indicate the progression of glaucoma. In linear regression analysis, BAI score (B = 0.058, 95% confidential interval = 0.020–0.097, *p* = 0.003) were significantly related to the rate of RNFL thinning, based on multivariate analyses. Figure 1 shows a significant positive correlation between the rate of RNFL thinning and BAI score. IOP parameters (mean, peak and fluctuation) were higher in the HA-G than the LA-G and DH occurred more often in the HA-G than the LA-G. Elevated IOP and disc hemorrhage are well-known risk factors for the development and progression of glaucoma. The results of our study indicate that anxiety is probably associated with variation in IOP and the occurrence of DH. IOP is one of the mechanical risk factors and DH is one of the vascular risk factors that indicate blood flow insufficiency. Faster progression in HA-G can probably be explained by these mechanical and vascular risk factors.

The VF MDs were worse in the HD-G (− 7.30 ± 7.68 dB) than the LD-G (− 3.81 ± 4.50 dB). This result suggests that the more severe a patient’s glaucoma is, the more likely they are to be depressed, which is consistent with previous reports^9,10^. The rate of RNFL thinning in the HD-G was faster than in the LD-G (*p* = 0.074, independent t-test), but with borderline statistical significance. The relationship with depression is weaker than that with anxiety, but suggests the possibility of an association with progression.

How could emotions such as anxiety and depression change mechanical (IOP) and vascular (DH) factors? There are several studies on the association between stress and iop^32,33^. They reported that psychological stress elevate IOP and cortisol hormone (known as the HPA axis) mediate this mechanism^32,33^. But hypothalamus first activates autonomic nervous system before it affects the hypothalamic–pituitary–adrenal (HPA) axis^34^. Anxiety and/or depression is a reaction to stress. When the body experiences a stressful event, the amygdala, an area of the brain that contributes to emotional processing, sends a signal to the hypothalamus^35^. The hypothalamus activates the adrenal medulla and causes the ‘fight or flight’ response via the sympathomedullary pathway^35^. The adrenal medulla, part of the ANS, secretes adrenaline, a hormone of fear. The ANS comprises the sympathetic nervous system (SNS) and the parasympathetic nervous system (PNS)^36^. When the body is stressed, the SNS contributes to coping with the threat^35^. SNS hormones increase the heart rate and respiration rate, and dilate blood vessels in the arms and legs to deal with the emergency^35,36^. When this reaction is over, the body usually returns to the pre-emergency, unstressed state^35,36^. This recovery is facilitated by the PNS, which generally has opposing effects to the SNS^35,36^. However, excessive PNS activity can also contribute to stress reactions such as bronchoconstriction, exaggerated vasodilation and compromised blood circulation^35,36^. Repetitive emotional changes and continual anxiety responses can destroy the balance in the ANS^36^. As the ANS is responsible for biological equilibrium in the body, it functions in regulation of the intraocular pressure and blood flow.

Although the relative importance of the mean, peak and fluctuation remain controversial, IOP is considered the most important modifiable factor in the development or progression of glaucoma^23–27^. Decreased ocular blood flow is associated with glaucoma progression^37^. DH, a surrogate for local blood flow disturbance, is also a well-known risk factor for the development and progression of glaucoma. Dysfunction of the ANS may impair all of these functions. In this way, emotional stress, such as anxiety or depression, affects the variation of IOP and the disturbance of blood flow through the unstable ANS.

There are limitations of the present study. First, since the questionnaire on anxiety or depression was measured at the time of study inclusion, it was not possible to confirm whether the scores were the same throughout the follow up period. Second, VF test could have fluctuation of accuracy, and anxiety could affect the reliability of VF. Third, glaucoma progression is slow, the observation period may not have been long enough. The observation period was not constant and the standard deviation was rather large in study. However, when comparing the two groups (high or low in anxiety or depression), these difference were not statistically significant. Finally, the small effects of other variables, confounding variables could have been fully apparent in the present analysis, because this study only included modest sample sizes.

To summarize, patients with anxiety showed faster rates of RNFL decline, as measured by OCT. These finding offer new insights into the care of patients with glaucoma. Therefore, the management of depression or anxiety may be helpful in managing glaucoma.

## Materials and methods

### Subjects

This study included 251 patients with open angle glaucoma who visited the Seoul St. Mary's Hospital between December 2018 and February 2020. It was approved by the Institutional Review and Ethics Board of Seoul St. Mary's Hospital. We followed the tenets of the Declaration of Helsinki. Informed consent was obtained from all the eligible subjects.

Only those patients with at least 2 years of follow-up were eligible for the study. One eye per patient was enrolled. If both eyes are eligible, only the right eye has been enrolled. All patients enrolled underwent ophthalmologic examination consisting of slit lamp biomicroscopy, IOP measurement, by Goldmann applanation tonometry; anterior chamber angle measurement, by gonioscopy; dilated stereoscopic examination of the optic disc; red-free fundus photography (Kowa nonmyd WX; Kowa Company Ltd., Tokyo, Japan); central corneal thickness, measured with ultrasound pachymetry (Tomey Corp, Nagoya, Japan); and axial length measurement, by ocular biometry (IOLMaster, Carl Zeiss Meditec, Dublin, CA). Retinal nerve fiber layer (RNFL) thickness was measured by the Cirrus OCT (Carl Zeiss Meditec). It calculates the global RNFL thickness automatically. Humphrey visual fields (VFs) were tested using Swedish Interactive Threshold Algorithm standard 24-2 perimetry (Carl Zeiss Meditec) at each visit. History of DH was investigated through review of medical records.

Open angle glaucoma (primary open angle glaucoma or normal tension glaucoma) was defined as the open angle, glaucomatous optic nerve damage, and associated, repeatable VF damage. Diagnosis of glaucomatous optic nerve damage was based on the presence of focal or diffuse thinning of the RNFL. Glaucomatous VFs were defined as a cluster of 3 or more non-edge points on the pattern deviation map with a probability < 5% of the healthy population, including at least 1 of those points with the probability of < 1% of the healthy population (reliable tests; fixation losses < 20%, false negative < 15% and false positives < 15%)^38^.

Optic disc tilt, torsion, and peripapillary atrophy (PPA)-to-disc ratio were measured on photographs, by two independent examiners (DYS and HYP) using image-analysis software (ImageJ version 1.40; http://rsb.info.nih.gov/ij/index.html; National Institutes of Health, Bethesda).

Optic disc tilt was defined as the ratio between the longest and shortest diameters of the optic disc^20,39^. Optic disc torsion was defined as the deviation of the long axis of the optic disc from the vertical meridian^20,40^. PPA-to-disc ratio was defined as the ratio between the PPA area and disc area (PPA to disc ratio = PPA area ÷ disc area)^20,41^. The areas of the PPA and disc were calculated using the imageJ software. The techniques for assessing the disc tilt, torsion and PPA-to-disc ratio have been described and applied in previous investigations^20^.

Mean IOP is the average of all measurements obtained during the follow up period. Peak IOP was the maximum IOP of all measurements obtained during follow-up period. The fluctuation of IOP was calculated by subtracting the lowest value from the largest value of the IOPs of all measurements obtained during follow-up period.

BP measurements included systolic and diastolic BP at the height of the heart, measured with an Omron Automatic BP instrument (model BP791IT; Omron Hearlthcare, Inc., Lake Forest, IL). Mean arterial BP was calculated as 1/3 systolic BP + 2/3 diastolic BP.

Heart rate variability was measured with a Medicore Heart rate Analyzer, Model SA-3000P (Medicore, Seoul, Korea). The standard deviation value of the qualified normal to normal intervals(SDNN) was used as a representative indicator of heart rate variability. It is believed to primarily be a measure of autonomic influence on heart rate variability.

### Beck’s Anxiety Inventory (BAI) and Beck’s Depression Inventory-II (BDI-II)

We used the BAI and BDI-II to evaluate psychological status^42,43^. The BAI and BDI-II are commonly used self-report questionnaires, used to determine the presence of anxiety disorder or depression disorder. We used these questionnaires measure common somatic and to evaluate the degree of anxiety or depression. The BAI questionnaire measures common somatic and cognitive symptoms of anxiety. Both the BAI and BDI-II include 21 items scored from 0 to 3, to generate a total score ranging from 0 to 63. Higher scores indicate greater anxiety/depression. In the BAI, total scores of 0–9 indicate normal levels of anxiety, and scores higher than 9 indicate clinically significant anxiety symptoms, based on published guidelines^42^. In the BDI-II, total scores higher than 13 indicate clinically significant depressive symptoms, based on guidelines and previous studies^44^. The questions on the BAI and BDI-II are listed in Tables 4 and 7, respectively.

### Statistical analysis

Sample size calculations were performed using a statistical power analysis program (G*Power 3.1 software). The minimum sample size was calculated as 244 total, 41 in group 1 and 203 un group 2 after setting the effect size at 0.05 (minimum size), the alpha error at 0.04 and the power at 0.80 (5 times difference between the two groups for t-test statistic).

To explore the hypothesis that in glaucoma patients, the group with high anxiety or depression will show different characteristics of glaucoma, patients were grouped and compared according to their BAI or BDI-II scores (separately). The independent t-test and chi-square test for independent samples were used to assess the differences between high and low (anxiety or depression) group. The RNFL loss rate was calculated from serial OCT measurements and observation times. Logistic regression analyses were used to identify parameters of the glaucoma that were associated with anxiety and depression. Factors with a *P*-value of < 0.05 in the univariate model were included in the multivariate model. *P*-values < 0.05 indicated statistical significance. All statistical analyses were performed with SPSS for Windows statistical software (ver.24.0; SPSS Inc., Chicago, IL). Data are presented as mean standard deviation except where stated otherwise. Linear regression analysis was used to search for correlations between the RNFL loss rate or MD of VF and the ocular parameters, including age, sex, and axial length, BAI score, BDI-II score, HRV and so on.
